# Prognostic nomogram of hypoxia-related genes predicting overall survival of colorectal cancer–Analysis of TCGA database

**DOI:** 10.1038/s41598-018-38116-y

**Published:** 2019-02-12

**Authors:** Joon-Hyop Lee, Sohee Jung, Won Seo Park, Eun Kyung Choe, Eunyoung Kim, Rumi Shin, Seung Chul Heo, Jae Hyun Lee, Kwangsoo Kim, Young Jun Chai

**Affiliations:** 1 Department of Surgery, Gachon University College of Medicine, Gil Medical Center, Incheon, Republic of Korea; 2 Division of Clinical Bioinformatics, Biomedical Research Institute, Seoul National University Hospital, Seoul, Republic of Korea; 3 Department of Surgery, Kyung Hee University Hospital, Seoul, Republic of Korea; 4 Department of Surgery, Seoul National University Hospital Healthcare System, Gangnam Center, Seoul, Republic of Korea; 5 Department of Surgery, National Medical Center, Seoul, Republic of Korea; 6 Department of Surgery, Seoul Metropolitan Government, Seoul National University Boramae Medical Center, Seoul, Republic of Korea; 7 Department of Statistics, Korea University, Seoul, Republic of Korea

## Abstract

Hypoxia-related gene (HRG) expression is associated with survival outcomes of colorectal cancer (CRC). Our aim was developing a nomogram predicting CRC overall survival (OS) with HRGs and clinicopathological factors. The Cancer Genome Atlas (TCGA) database was used as discovery cohort and two Gene Expression Omnibus databases (GSE39582 and GSE41258) served as validation cohorts. A genetic risk score model prognosticating OS was developed using mRNA expression level of HRGs. Nomogram predicting OS was developed using genetic risk score model and clinicopathological variables. The genetic risk score model included four HRGs (*HSPA1L*, *PUM1*, *UBE2D2*, and *HSP27*) and successfully prognosticated OS of discovery and two validation cohorts (*p* < 0.001 for TCGA discovery set, *p* < 0.003 for the GSE39582 and *p* = 0.042 for the GSE41258 datasets). Nomogram included genetic risk score, age, and TNM stage. Harrell’s concordance indexes of the nomogram were higher than those of TNM stage alone in the discovery set (0.77 vs. 0.69, *p* < 0.001), GSE39582 (0.65 vs. 0.63, *p* < 0.001), and GSE41258 datasets (0.78 vs. 0.77, *p* < 0.001). Our nomogram successfully predicted OS of CRC patients. The mRNA expression level of the HRGs might be useful as an ancillary marker for prognosticating CRC outcome.

## Introduction

Globally, colorectal cancer (CRC) is the second most common cause of cancer related mortality and the fourth most frequently diagnosed malignancy^1^. Treatment plans and clinical outcomes of CRC are primarily based on well documented conventional clinicopathologic risks and prognostic factors such as age, tumor stage, diet, alcohol consumption or smoking etc^2,3^. With the recent progress in genetic profiling including microsatellite instability, molecular signature, and oncogene analysis, new prognostic data for treatment of CRC have now become more diverse^4,5^.

Hypoxic tumor microenvironments are associated with poor outcomes and survival^6,7^. Hypoxic foci are formed when cancer cell metabolic requirements surpass the intravascular oxygen availability of a tumor. Genes whose expression changes are triggered under such conditions are referred to as hypoxia-related genes (HRG)^7^. Their prognostic abilities on outcome of major malignancies such as breast or gastric cancer have been well documented^8,9^. Prolific research has been conducted on the prognostic and predictive values of molecular profiles associated with hypoxia and CRC survival outcomes^7,10–12^.

Although the role of HRG expression on outcome prediction in CRC has been demonstrated, most studies lack systematic methodology and focus primarily on separate gene expression and its correlation with CRC outcomes regardless of the clinical setting^13–17^. The aim of this study was to formulate a nomogram to predict overall survival (OS) of CRC using a genetic risk score which is based on the mRNA expression level of HRGs, as well as clinicopathological variables.

## Results

### Baseline characteristics

The discovery TCGA cohort consisted of 355 patients who were diagnosed with CRC at a mean age of 64.5 years and followed-up for a median and mean interval of 22 months (0–148 months) and 31 months, respectively. The validation GSE39582 cohort consisted of 557 patients who were diagnosed at a mean age of 66.8 years and followed-up for a median and mean interval of 52 months (0–201 months) and 57 months, respectively. The validation GSE41258 cohort included 185 patients who were diagnosed at a mean age of 63.5 years and followed-up for a median and mean interval of 66 months (0–203 months) and 68 months, respectively (Table 1).

**Table 1:** Patient characteristics of datasets.

Discovery cohort		Validation cohort		Validation cohort	
TCGA (n = 355)		GSE39582 (n = 557)		GSE41258 (n = 185	
Characteristic	n (%)	Characteristic	n (%)	Characteristic	n (%)
Age at diagnosis		Age at diagnosis		Age at diagnosis	
Mean (SD)	64.5 (13.3)	Mean (SD)	66.8 (13.3)	Mean (SD)	63.5 (14.0)
AJCC TNM stage		AJCC TNM stage		AJCC TNM stage	
I	56 (15.8)	I	31 (5.6)	I	28 (15.1)
II	135 (38.0)	II	262 (47.0)	II	50 (27.0)
III	112 (31.5)	III	204 (36.6)	III	49 (26.5)
IV	52 (14.7)	IV	60 (10.8)	IV	58 (31.4)
Survival event		Survival event		Survival event	
Dead	78 (22.0)	Dead	190 (34.1)	Dead	102 (55.1)
Alive	277 (78.0)	Alive	367 (65.9)	Alive	83 (44.9)
Median follow-up time, months (range)		Median follow-up time, months (range)		Median follow-up time, months (range)	
	22 (0–148)		52 (0–201)		66 (0–203)
Median time to survival event, months (range)		Median time to survival event, months (range)		Median time to survival event, months (range)	
	17 (1–100)		31 (0–183)		34 (0–196)

TCGA: The cancer genome atlas; GEO: gene expression omnibus; SD: standard deviation; AJCC: American Joint Committee on Cancer.

### Genetic risk score model construction

**Figure 1:**
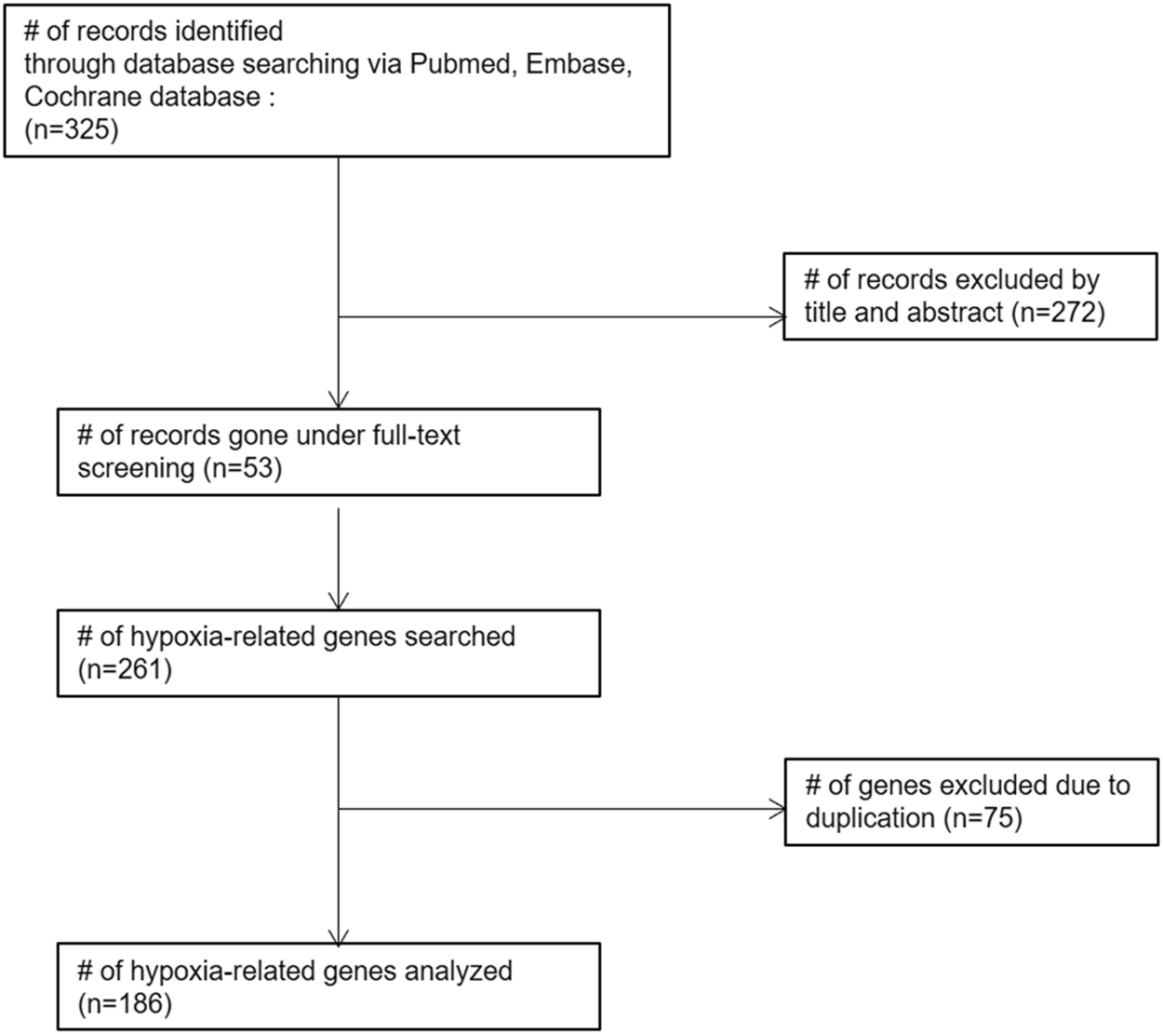
Gene selection flow chart.

**Figure 2:**
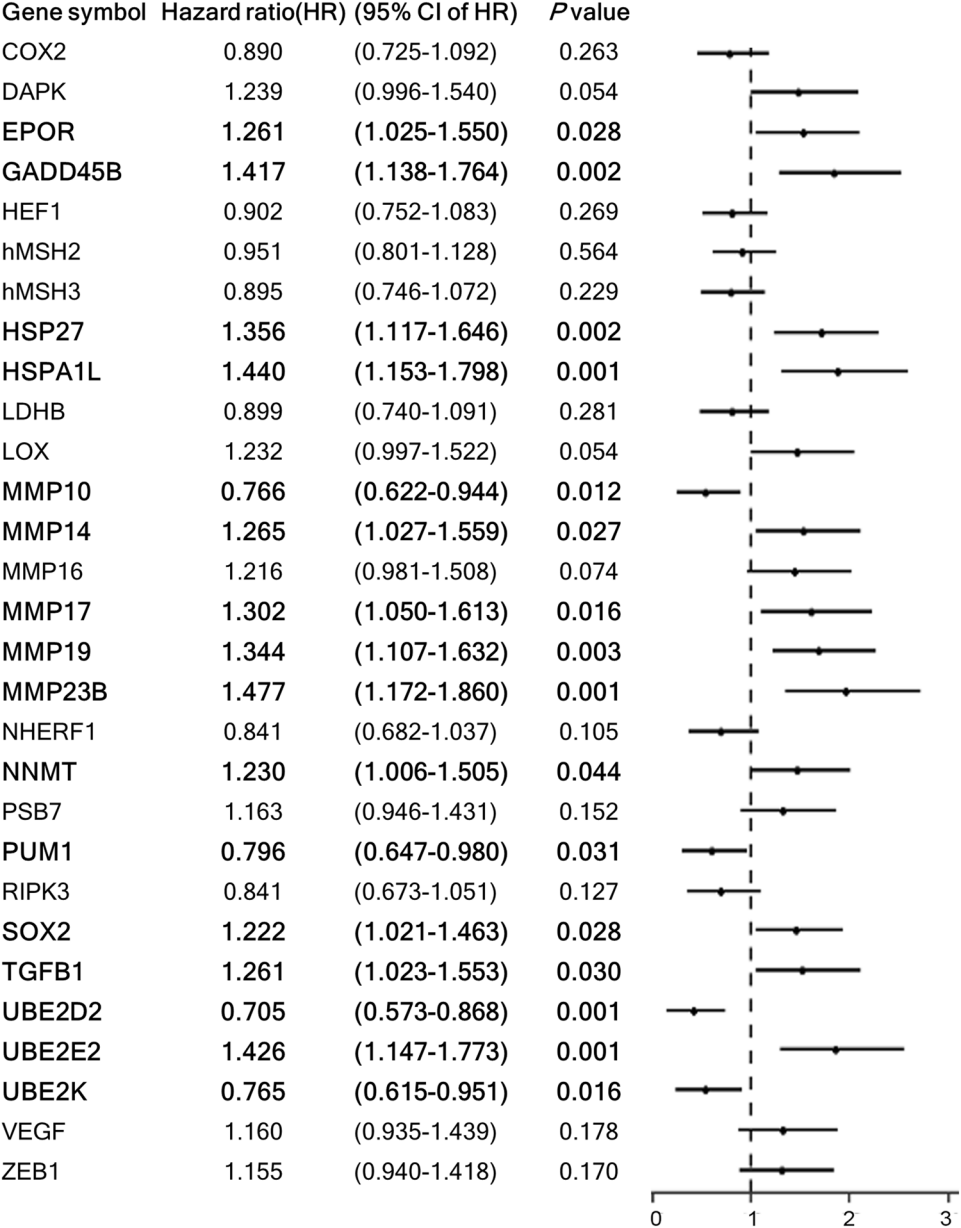
Twenty-nine hypoxia-related genes which were significantly associated with OS by log-rank test. Among them, 16 genes were associated with overall survival in univariate analysis and are highlighted in bold.

Among the 325 publications searched, 53 articles relevant to CRC gene-expression in hypoxic conditions were reviewed. One hundred and eighty-six genes were selected from the reviewed articles (Fig. 1). Twenty-nine HRGs were significantly associated with OS by log-rank test, and their relationship with OS was further investigated by univariate Cox regression, which demonstrated 16 genes to be associated with OS (Fig. 2).

**Figure 3:**
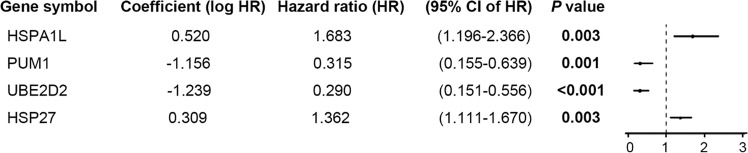
Genetic risk score model developed with 4 hypoxia-related genes.

Pairwise Pearson correlation coefficients among the 16 genes revealed two gene groups that were closely related amongst each other among which the one with the highest hazard ratio (HR) was selected; *EPOR* and *TGFB1* groups (HR for *EPOR*, 1.2608, was higher), and *MMP23B*, *MMP14*, *MMP17*, *MMP19*, *NNMT*, *TGFB1*, *UBE2E2* cluster (*MMP23B* had the highest HR of 1.4766). And other genes that were not closely associated among each other (*UBE2K*, *HSPA1L*, *HSP27*, *MMP10*, *SOX2*, *UBE2D2*, *PUM1*) were included in the stepwise Cox regression analysis, resulting in the following genetic risk score model:

Genetic risk score = 0.520*HSPA1L −1.156*PUM1 −1.239*UBE2D2 + 0.309*HSP27 (Fig. 3).

### Prognostic value of genetic risk score

**Figure 4:**
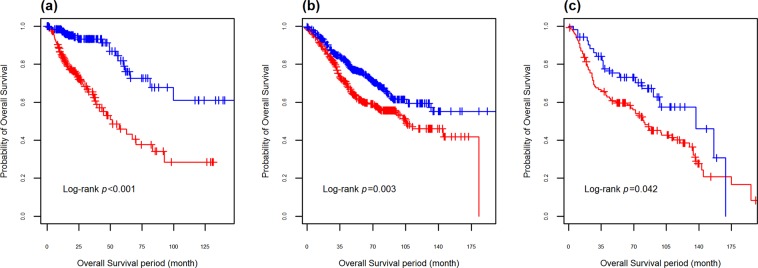
Kaplan-Meier plot of the genetic risk score (high risk vs. low risk, threshold: median score) for (**a**) TCGA discovery set, (**b**) GSE39582 validation set, and (**c**) GSE41258 validation set.

The genetic risk score was categorized at the optimal cutoff point (high risk vs. low risk) based on the receiver operating characteristics (ROC) curve. The prognostic ability of the genetic risk score model was demonstrated by the significant difference between the survival curves of the high risk and low risk group observed in both discovery (TCGA) and validation (GSE39582, GSE41258) cohorts (*p* < 0.001, *p* = 0.003 and *p* = 0.042) (Fig. 4).

### Incorporating clinical factors to predict cancer survival

The genetic risk score (high risk vs. low risk) was associated with OS in the univariate analysis (*p* < 0.001). After statistical adjustment for other variables with multivariate Cox analysis, the genetic risk score, TNM stage, and age were independently prognostic of OS (Table 2).

**Table 2:** Univariate and multivariate Cox-regression results of factors related to overall survival.

Univariate analysis				Multivariate analysis			
Variables	Hazard Ratio (95% CI)		*P* value	Variables	Hazard Ratio (95% CI)		*P* value
Genetic risk score (High risk vs. low risk)	4.221	(2.536–7.026)	<0.001	Genetic risk score (High risk vs. low risk)	3.402	(2.873–3.93)	<0.001
Age	1.025	(1.007–1.044)	0.006	Age	1.029	(1.009–1.049)	0.004
Male gender	1.264	(0.805–1.985)	0.307	Male gender	1.062	(0.598–1.527)	0.798
AJCC TNM stage			<0.001	AJCC TNM stage			<0.001
I	1.000 (reference)			I	1.000 (reference)		
II	1.450	(0.547–3.845)		II	1.057	(0.066–2.048)	
III	2.820	(1.090–7.293)		III	2.082	(1.115–3.049)	
IV	6.511	(2.461–17.225)		IV	5.733	(4.739–6.726)	
*KRAS* mutation	0.797	(0.505–1.257)	0.325	*KRAS* mutation	0.805	(0.31–1.3)	0.389
*BRAF* mutation	1.257	(0.692–2.285)	0.464	*BRAF* mutation	1.327	(0.504–2.151)	0.508
MSI-high	0.800	(0.422–1.515)	0.481	MSI-high	0.759	(−0.106–1.6)	0.526

AJCC: American Joint Committee on Cancer; MSI: microsatellite instability.

A set of prognostic models for OS was constructed by combining the genetic risk score (high risk vs. low risk), TNM stage, and age into the multivariate Cox regression model.

In the TCGA discovery set, Harrell’s concordance index (C-index) for the model which included TNM stage and genetic risk score was higher than that of TNM stage alone (0.75 vs. 0.69, *p* < 0.001). C-index for the model including age, TNM stage, and genetic risk score was higher than that of the model which included TNM stage and genetic risk score (0.77 vs. 0.75, *p* < 0.001).

In the GSE39582 validation set, C-index for the model which included TNM stage and genetic risk score was higher than that of TNM stage alone (0.65 vs. 0.63, *p* < 0.001). C-index for the model which included age, TNM stage, and genetic risk score was higher than that of the model which included TNM stage and genetic risk score (0.70 vs. 0.65, *p* < 0.001).

In the GSE41258 validation set, C-index for the model which included TNM stage and genetic risk score was higher than that of TNM stage alone (0.78 vs. 0.77, *p* < 0.001). C-index for the model which included age, TNM stage, and genetic risk score was same as that of the model which included TNM stage and genetic risk score (0.78 for both).

### Nomogram including genetic risk score and clinical attributes

**Figure 5:**
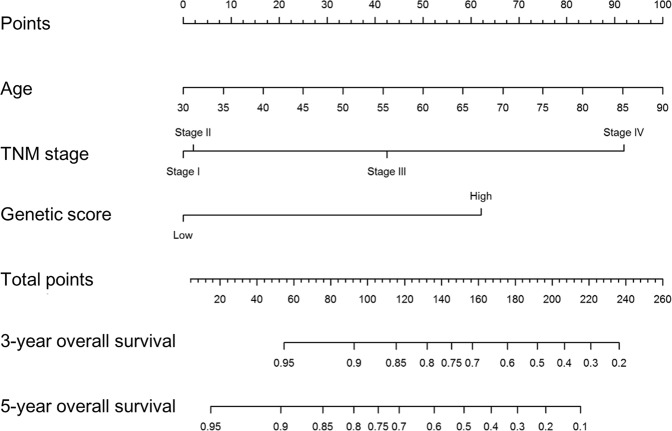
Nomogram predicting 3- and 5-year overall survival of colorectal cancer patients.

**Figure 6:**
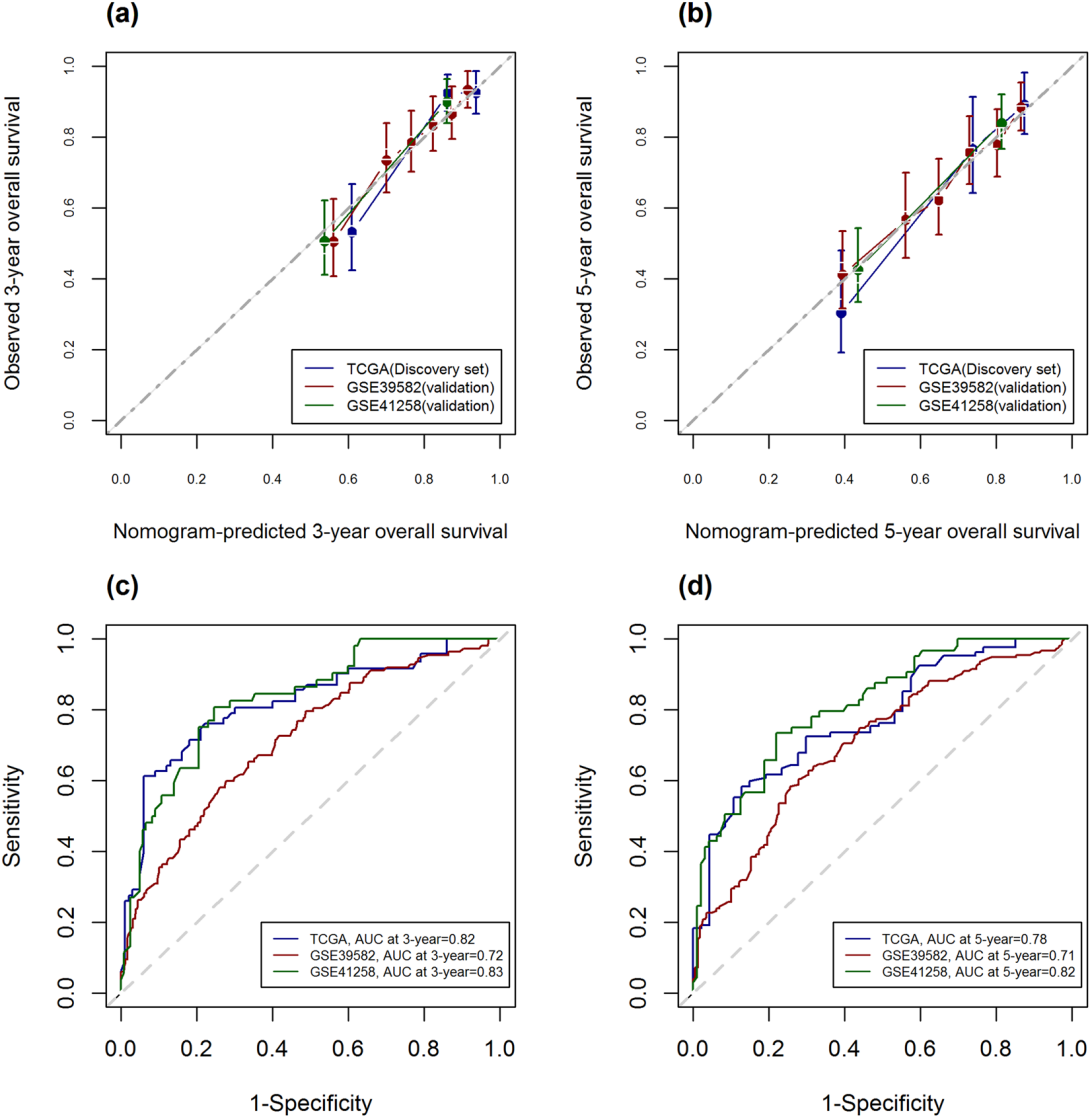
Calibration curve for nomogram-predicting (**a**) 3-year and (**b**) 5-year overall survival. The X-axis is nomogram-predicted survival probability and the Y-axis is observed survival probability respectively. Red, green and blue solid lines represent the performance of the nomogram relative to the 45-degree line, indicating perfect prediction. Receiver operating characteristic curves assessing the discriminating ability of the nomogram in predicting (**c**) 3-year and (**d**) 5-year overall survival.

Based on the C-index values, a nomogram integrating the genetic risk score (high risk vs. low risk), age, and TNM stage was constructed (Fig. 5). Total points were calculated by adding the points of the genetic score, age, and TNM stage. The calibration curve for predicting 3 and 5-year OS indicated that the nomogram-predicted survival closely corresponded with actual survival outcomes. The 3-year nomogram’s area under curve (AUC) was 0.82 in the TCGA discovery set, 0.72 in the GSE39582 and 0.83 in the GSE41258 cohort. The 5-year nomogram’s AUC was 0.78 in the TCGA cohort, 0.71 in the GSE39582 and 0.82 in the GSE41258 cohort. (Fig. 6).

## Discussion

This study is the first to construct a nomogram of CRC OS that encompasses both clinical attributes and effect of HRGs quantified by a risk score system. Our genetic risk score and nomogram’s CRC prognostic ability was proven to be superior to conventional TNM stage for predicting prognosis in both the discovery TCGA cohort and the validation GSE39582 and GSE41258 datasets.

Hypoxia is a common feature in malignancy that promotes invasive and metastatic tumor behavior^18^. Expression of HRG is involved in cellular processes such as differentiation, angiogenesis, survival, migration, and metastasis^19^. In breast cancer, analysis of HRGs has been proposed as a tool for developing novel therapeutic strategies with molecular signatures^20^. The prognostic ability of HRGs is reported in many other malignancies, including gastric cancer, leukemia, and CRC^8,21,22^.

Our genetic risk scoring model was based upon a combination of *HSPAL1L*, *PUM1*, *UBE2D2*, and *HSP* gene mRNA expressions that were selected from among 186 HRGs to quantitatively predict the prognosis of CRC. The heat-shock 70-kDa protein-1-like (*HSPA1L*) gene is pivotal in tumor niche condition-induced *HIF-1α* activation and cellular prion protein (PrP^C^) regulation and leads to CRC proliferation^23^. The ubiquitin conjugating enzymes E2 (*UBE2)* gene family prevents HIF1α and 2α degradation by proteasome systems, and *UBE2* inhibitors act as antitumor agents^24^. Abnormal pumilio RNA binding family member 1 (*PUM1*) gene expression is closely related to carcinogenesis and chromosomal mutations^25^, and heat shock protein 27 (*HSP27*) expression has a protective effect on hypoxic injury related umbilical cord blood-derived mesenchymal stem cell apoptosis^26^. Although the most researched HRG is the *HIF* (hypoxia-inducible factor) gene family which is important in mediating response to hypoxia at the cellular level^27,28^, the TCGA database indicated that mRNA expression levels of the *HIF1A*, *HIF1B*, *HIF2A*, and *HIF3A* genes were not statistically associated with OS and DFS (data not shown).

There are many published nomograms designed to predict the outcome of CRC^29^. A Chinese group developed a nomogram on CRC OS and recurrence-free survival for stage I~III patients^30^, and a French group targeted metastatic stage IV CRC patients who were refractory to chemotherapy^31^. The C indexes of these studies were 0.80 and 0.7, respectively. However, there is no single nomogram that encompasses the long-term OS outcome of all clinical stages of CRC. Ours is the first to included basic clinical variables integrated with a genetic risk score model of selected HRGs across all CRC stages.

The strength of this study was that we established validation sets of heterogenous patients from the GSE39582 and GSE41258 dataset to validate the generalizability of our genetic risk score model and nomogram. We believe this approach has important clinical implications because we validated the prognostic ability of the genetic risk score model and nomograms using mRNA data produced through different platforms. Discovery TCGA data was produced by RNA sequencing using the Illumina HiSeq. 2000 mRNA-Seq and the validation sets (GSE39582 and GSE41258) mRNA expression profiles were acquired by the Affymetrix microarray. Similar comparison of mRNA gene expression through different platforms in the literature further strengthens the generalizability of our results^32,33^.

There are several limitations to our study. One is the short follow-up period of the discovery set patients. The median follow-up period of the discovery set, which the gene risk model and nomogram was built on, was 22 months. To address the issue of the short follow-up duration, we formulated a nomogram based on both the 3-year and 5-year survival rates to better fit the median follow-up period. Another limitation is that mRNA gene expression values are not readily available especially in clinical settings due to the high cost of fresh tissue storage and processing. However, its applicability may become wider when costs decrease and mRNA expression can be stably obtained through formalin-fixed paraffin-embedded tissue. The final limitation is the inability to adjust for confounders pertaining to lifestyle factors, such as diet or smoking, operative extent and treatment modality. We could not account for these factors because TCGA and GEO databases do not provide information on them.

In conclusion, our study is the first to construct a nomogram for all stages of CRC OS encompassing both clinical and genetic variables related to HRGs. Our genetic risk score and nomogram demonstrated superior prognostic ability for CRC OS in all of the TCGA discovery and two external validation sets compared to conventional TNM staging. Considering the effect HRGs have on survival outcomes of CRC patients, our results may be applicable in the clinical setting in the near future. A more precise 5-year OS nomogram could be obtained by expanding the record duration of the discovery dataset patients with additional follow-up and subsequent modifying of our present results.

## Methods

### Data sources and processing

Gene mRNA expression data and related clinical information of CRC patients in the TCGA project (discovery cohort) were obtained from the CBioPortal (http://www.cbioportal.org). The mRNA-Seq data from TCGA was produced using the Illumina HiSeq 2000 platform and processed by the RNAseqV2 pipeline, which uses MapSplice for alignment and RSEM for quantification. To validate the prognostic potential of the genetic risk score, two independent datasets were obtained through the GEO database (GSE39582, GSE41258) (validation cohort, http://www.ncbi.nlm.nih.gov/geo/). Keywords “colorectal cancer” and “gene expression” were used for searching. Datasets satisfying the following criteria were considered: (1) gene expression profile data, (2) tissue samples from primary colorectal adenocarcinoma, and (3) availability of patient survival data. The GSE39582 and GSE41285 datasets, containing the largest and the second largest samples among those satisfying our criteria, were used for validation^34,35^. Gene expression profiles of the dataset were determined using the Affymetrix U133 Plus 2.0 chip. GSE39582 contained log2 signal intensity values and the gene expression levels of the TCGA and GSE41258 dataset were transformed to log2 scale. The median duration of record length (henceforth mentioned as follow-up period) was described in months. Information about CRC stage of both datasets was assessed according to the TNM stages specified by the 8^th^ edition of the American Joint Committee on Cancer^36^. To prevent the clinical data from becoming too specific the sub-stages were not assigned.

### Genetic risk score model construction

A qualitative review of literature related to CRC was conducted through the PubMed/MEDLINE database, using the following advanced search combination: (Colon OR Rectum OR Colorectal) AND (Cancer OR Neoplasm) AND Hypoxia AND Gene. Articles with relevant titles were fully reviewed for information about genes analyzed in hypoxic conditions to assess the outcome of CRC. Based on the literature, we selected appropriate genes for further analysis and construction of a genetic risk score. Among closely correlated genes (Pearson correlation coefficient *r* > 0.4), those with highest univariate predictive power (defined by HR per 1 standard deviation change) were selected to avoid potential collinearity^37,38^. To build the genetic risk score model, genes whose expression levels were significantly associated with OS were further selected through stepwise Cox regression analysis^39^. In the stepwise procedure, *p* < 0.05 was used as entry criterion and *p* > 0.1 as removal criterion^40^. The prognostic value of the genetic risk score model was assessed with both discovery and validation cohorts. The optimal cut-off point for the genetic risk score was determined based on ROC curve analysis. Hypoxia-related activities of the selected genes were confirmed using the gene ontology database (http://www.geneontology.org/).

### Incorporating clinical factors to predict cancer survival

To evaluate the prognostic value of the genetic risk score in the context of other clinical variables, univariate and multivariate Cox analyses for OS were performed, including the genetic risk score and the conventional clinicopathologic variables (age, gender, TNM stage, *KRAS* mutation, *BRAF* mutation, and microsatellite instability). The discriminating ability of the multivariate Cox regression model was evaluated using the C-index^41^ of 1 indicating perfect discrimination and of 0.5 indicating random guess.

### Nomogram construction

A nomogram was constructed to predict 3- and 5-year CRC OS by combining the results of the genetic risk score model with clinical attributes. The predictive accuracy of the nomogram was assessed by calibration plot^42,43^. Time-dependent sensitivities and specificities of the nomogram were evaluated by AUC for both 3-year and 5-year OS ROC curve^44^. All statistical analyses were performed using R statistical software (version 3.4.1)^45^. Nomogram and calibration plots were generated with the rms package^46^ and the time-dependent ROC curve analysis was conducted with the timeROC package^47^ of R software. Comparisons of C-index between the nomogram and American Joint Committee on Cancer staging systems were performed with the Hmisc package^48^ of R software. Null hypotheses of no difference were rejected if *p*-values were less than 0.05.

## Data Availability

The data that support the findings of this study are available from the Cancer Genome Atlas (TCGA, http://cancergenome.nih.gov/) COADREAD project and Gene Expression Omnibus (GEO, https://www.ncbi.nlm.nih.gov/geo/), accession number GSE39582 and GSE41258.
